# HBsAg quantification predicts off-treatment response to interferon in chronic hepatitis B patients: a retrospective study of 250 cases

**DOI:** 10.1186/s12876-020-01263-6

**Published:** 2020-04-21

**Authors:** Shuai Wu, Wenfan Luo, Yin Wu, Hongjie Chen, Jie Peng

**Affiliations:** Department of Infectious Diseases, Nanfang Hospital of Southern Medical University, Guangzhou, 510515 China

**Keywords:** Interferon, Chronic hepatitis B, End-of-treatment, Hepatitis B surface antigen, Post-treatment response

## Abstract

**Background:**

For chronic hepatitis B (CHB) patients without willingness to extend the routine duration of interferon (IFN) therapy, it is important to identify patients who will benefit from treatment cessation. Hepatitis B surface antigen (HBsAg) quantification is recommended for management of IFN therapy. At present, the understanding on end-of-treatment (EOT) HBsAg level predicting post-treatment response to IFN is still finite.

**Methods:**

A total of 2451 non-cirrhosis, HBsAg-postive patients treated with IFN-based therapy during the period from December 2010 to December 2017 at Nanfang Hospital were enrolled in this study. Serum HBsAg levels at EOT were measured to evaluate the associations between EOT HBsAg levels (Group 1, HBsAg > 0.05 and ≤ 10 IU/mL; Group 2, HBsAg > 10 and ≤ 200 IU/mL; Group 3, HBsAg > 200 IU/mL) with post-treatment HBsAg loss. Chi-squared, t-test,,Kaplan-Meier analysis, Cox regression analysis, and Multivariate Logistic regression analysis were used to analyse and evaluate differences between the there groups.

**Results:**

The cumulative HBsAg loss rates 5 years after treatment in Group 1–3 were 30.4% (17/56), 9.8%(4/41) and 0%(0/153) (*p* < 0.001). An EOT HBsAg level of > 10 IU/mL showed relatively high negative predictive value (NPV) of up to 97.9% for HBsAg loss. Low baseline HBsAg level < 25,000 IU/mL, on-treatment HBsAg decline > 1 log10IU/mL at week 24 and EOT HBsAg level ≤ 10 IU/mL were found significantly associated with HBsAg loss. A total of 6 patients have achieved HBsAg loss at EOT and 17 patients with EOT HBsAg level ≤ 10 IU/mL have achieved post-treatment HBsAg loss. Baseline characteristics, dynamic changes of on-treatment HBsAg and duration of IFN therapy were balanced across patients with EOT or post-treatment HBsAg loss.

**Conclusion:**

EOT HBsAg level can serve as a monitoring indicator for IFN therapy. EOT HBsAg level ≤ 10 IU/mL was found to lead to high rate of post-treatment HBsAg loss. For patients without willingness to extend IFN treatment, off-treatment follow-up could be considered when HBsAg level decreased to ≤10 IU/mL.

## Background

Within World Health Organization regions, hepatitis B virus (HBV) infection remains a major public problem with approximately 2 billion people infected globally. Among these, 240 million people have been suffering from chronic HBV infection, and nearly 650,000 individuals died annually of HBV-induced liver failure, cirrhosis and hepatocellular carcinoma (HCC) [1–3].

To prevent disease progression, antiviral therapy is necessary. The main goal of therapy is to improve survival and quality of life by long-term suppression of viral replication, alleviating hepatic necroinflammation and fibrosis, and consequently reducing the risk of HCC development [4–8]. Recent guidelines recommend antiviral treatment with nucleos(t) ide analogues (NUCs) or with interferon-α (IFN-α) for chronic hepatitis B (CHB) patients. The main efficacy of NUCs is to inhibit HBV replication leading to undetectable HBV DNA levels, but hepatitis surface antigen (HBsAg) loss, representing a functional cure, is rarely achieved. Moreover, due to the high risk of relapse after NUCs discontinuation, long-term consolidation treatment is often required, leading to increasing risk of drug-related side effects and drug resistance. Interferon (IFN) provides a finite duration treatment by direct antiviral effects and immune modulation, long-term immunological control after treatment discontinuation is also induced to impede viral activity [9, 10]. It has been demonstrated by recent large randomised NEPTUNE study that the immune response of IFN therapy is durable for up to 5 years [11]. Since long-term benefits of CHB patients are held in highly regard, a five-year observational cohort study demonstrated that treatment with IFN leads to a significant lower incidence of unfavorable events than entecavir in CHB patients [12]. A phase 3 clinical trial has also reported that in hepatitis B e antigen (HBeAg)-positive CHB patients treated with IFN, 14% initial non-responders achieved delayed response 6–12 months post-treatment, and 86% initial responders maintained sustained response for up to 1 year [13].

Both Chinese and EASL guidelines recommend HBsAg quantification for management of IFN therapy [14, 15]. For HBeAg positive patients with HBsAg level < 200 IU/mL or HBeAg negative patients with HBsAg level ≤ 10 IU/mL at the end of IFN therapy, extended treatment is recommended [14]. However, for individuals, whether extended treatment will lead to HBsAg loss and how long the treatment will last, remain unknown. Thus, for CHB patients without willingness to extend IFN therapy, it is important to identify patients who will benefit from treatment cessation. Recent researches identified baseline HBsAg quantification and on-treatment dynamic changes of HBsAg as predictors for treatment response to IFN [16–21]. However, the understanding on HBsAg level predicting post-treatment response to IFN is still finite [22].

We thus conducted a retrospective study using the data of CHB patients treated with IFN-based therapy at Nanfang hospital (Guangzhou, China). The main aims of this study were (1) to investigate the association between end-of-treatment (EOT) HBsAg level and post-treatment HBsAg loss, (2) to identify factors associated with EOT HBsAg level or post-treatment HBsAg loss.

## Methods

### Study population

**Fig. 1:**
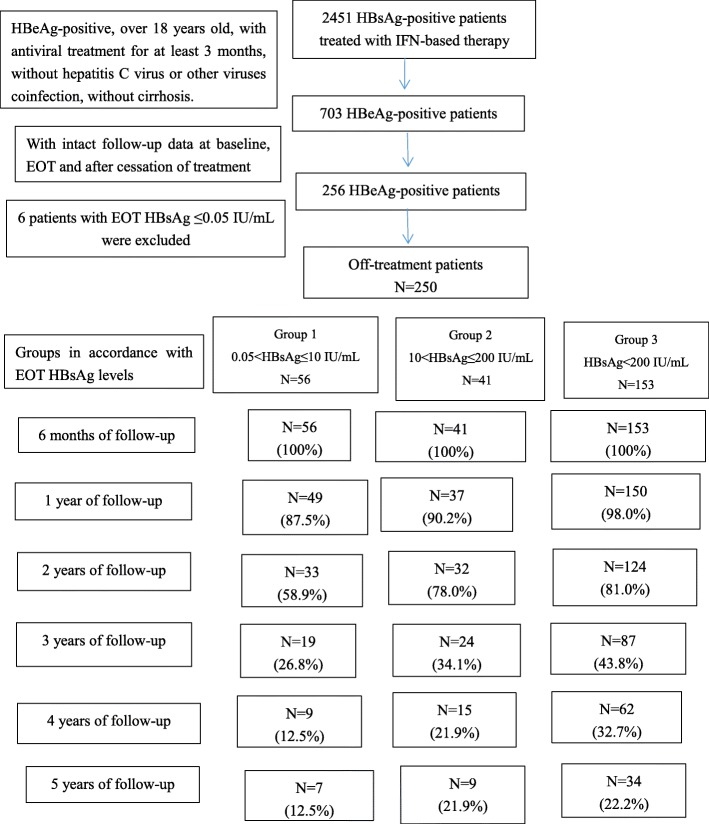
Flowchart of study. IFN, interferon. EOT, end-of-treatment

This was a retrospective study consisting of consecutive CHB patients who have received IFN-based therapy (standard IFN-alpha or peg-IFN alpha) during the period from December 2010 to December 2017 at Nanfang Hospital of Southern Medical University in Guangzhou, China. This hospital is a public-care, teaching-medical centre in Guangzhou that serves as a patient referral centre and accepts patient referrals from every part of Guangzhou. A total of 2451 HBsAg-positive patients were enrolled in this study. All patients had the same inclusion criteria: HBeAg-positive, over 18 years old, with IFN-based therapies only or combined with oral nucleos(t) ide analogues for at least 3 months, without hepatitis C virus or other viruses co-infection, without cirrhosis and with intact follow-up data at baseline, EOT and after cessation of treatment. The details of the follow-up were shown in Fig. 1. All patients were divided into three groups in accordance with EOT HBsAg levels: Group 1- HBsAg > 0.05 and ≤ 10 IU/mL (*n* = 56), Group 2- HBsAg > 10 and ≤ 200 IU/mL (*n* = 41), Group 3- HBsAg > 200 IU/mL (*n* = 153). The EOT HBsAg cut-off value selection was based on previous studies [22, 23].HBsAg loss was defined as serum HBsAg < 0.05 IU/mL.

### Data collection

Data were collected by reviewing the medical records of each patient. The records included demographic characteristics (age and sex), HBsAg levels, HBV DNA viral load, duration of IFN, laboratory values (ALT).

### Laboratory testing

Serum HBV DNA was tested with a polymerase chain reaction HBV assay with a lower limit of detection (LLOD) of 1000 copies/mL (Daan Gene Co, Ltd.; Sun Yat-sen University; Guangzhou, China). Serum HBsAg was quantified using the ARCHITECT HBsAg assay (range 0.05–250 IU/mL; Abbott Laboratories, Chicago, Il, USA).

### Statistical analysis

Continuous variables were shown as mean ± standard deviation (SD) and median (min-max) for data with gaussian distribution and skewed distribution, respectively. Intra-group differences were analyzed by t-test, when appropriate. Differences between groups were analyzed by Kruskal-Wallis H test and F-test, when applicable. Categorical variables were analyzed with Chi-squared test and shown as numbers (rate). We performed Kaplan-Meier analysis to compare cumulative HBsAg seroclearance rates between patients with different EOT HBsAg levels and data were censored after 5 years of follow-up. Cox regression analysis was conducted to identify prognostic factors for HBsAg loss in off-treatment patients. Multivariate Logistic regression analysis was also performed to identify potential factors associated with EOT HBsAg level. All statistical analyses were carried out with IBM SPSS Statistics for Windows, V.24.0. A *p*-value < 0.05 was taken for statistical significance.

## Results

### Patient characteristics

Baseline characteristics of all patients grouped according to EOT HBsAg levels were summarized in Table 1. Except for the duration of IFN and baseline HBsAg level, in the distribution of gender, age, baseline HBV DNA and ALT level, no significant differences were observed between the three subgroups.

**Table 1:** General characteristics of patients

	Group 1	Group 2	Group 3	*p-*value
	*N* = 56	*N* = 41	*N* = 153	
Gender, male (%)	44 (78.6%)	33 (80.5%)	113 (73.9%)	0.594
Age (years)	27.3 ± 6.3	29.9 ± 8.2	29.1 ± 6.9	0.147
Duration of IFN (months)	15.3 ± 5.8	17.6 ± 7.3	12.0 ± 6.0	***< 0.001***
Baseline HBsAg (log_10_IU/mL)	3.1 ± 1.3	3.1 ± 1.0	3.9 ± 0.6	***< 0.001***
Baseline HBV DNA (log_10_IU/mL)	5.7 ± 2.2	5.9 ± 1.9	6.2 ± 1.9	0.453
Baseline ALT (U/L)	154.1 ± 143.8	109.1 ± 106.9	169.2 ± 213.4	0.356

*IFN* interferon, *ALT* alanine aminotransferase. Continuous variables are shown as mean ± SD, categorical variables as n (%). *P*-values < 0.05 are shown in bold italics

### Correlation of EOT HBsAg level with post-treatment HBsAg loss

**Fig. 2:**
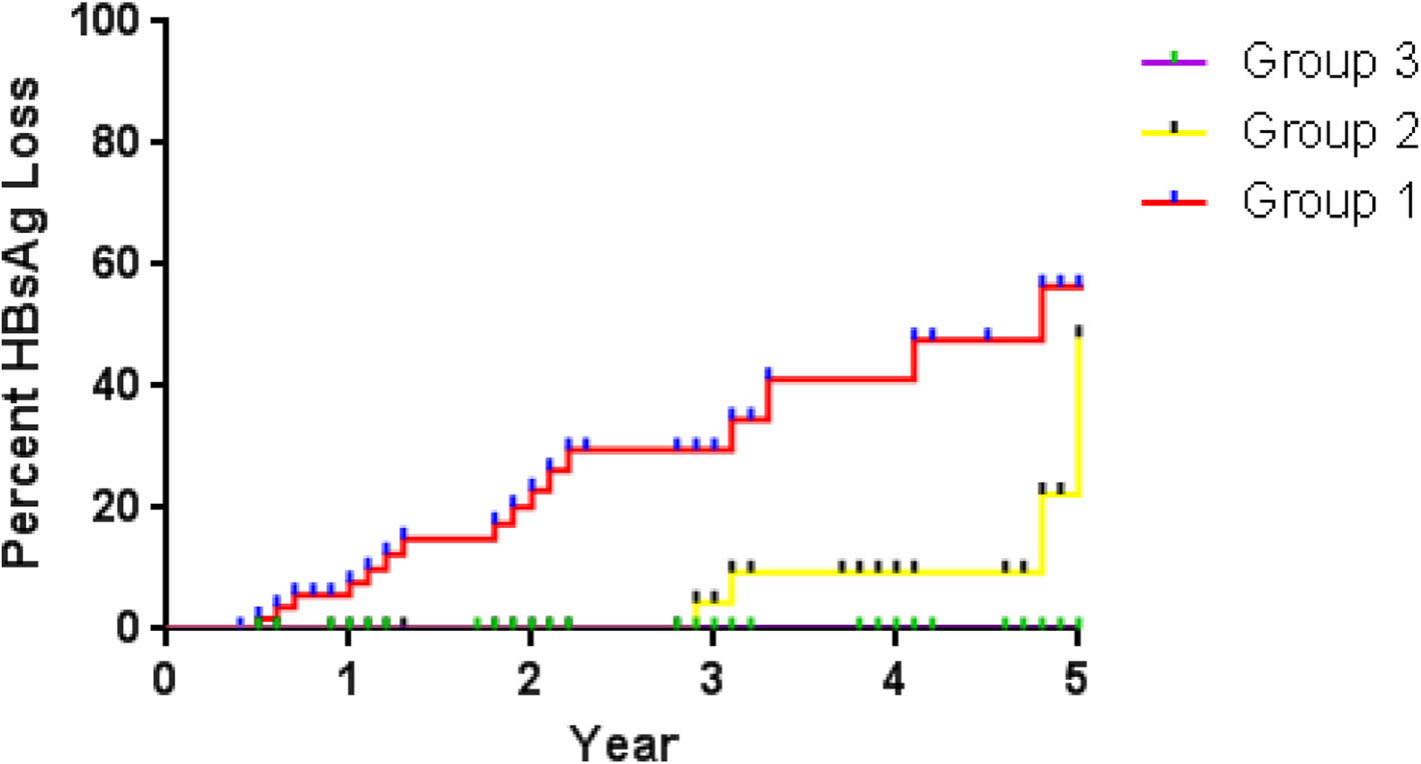
Association of EOT HBsAg level with cumulative HBsAg loss rates. Kaplan Meier curves show the association of EOT HBsAg level with cumulative HBsAg loss rates. Group 1, HBsAg > 0.05 and ≤ 10 IU/mL; Group 2, HBsAg > 10 and ≤ 200 IU/mL; Group 3, HBsAg > 200 IU/mL. The Kaplan Meier analysis obtained a *p*-value of < 0.001

At the EOT, the average expression of HBsAg in all patients was 2.4 ± 1.7 log10IU/mL. 22.4% patients had HBsAg level of > 0.05 and ≤ 10 IU/mL, 16.4% had HBsAg level of > 10 and ≤ 200 IU/mL, and 61.2% had HBsAg level of > 200 IU/mL. Kaplan-Meier analysis was conducted to evaluate the association of EOT HBsAg level with cumulative HBsAg loss rates. After 5 years of follow-up, the patients in Group 1 exhibited significantly higher HBsAg loss rate of 30.4% (17/56), and in group 2 and group 3, the cumulative HBsAg loss rates were 9.8% (4/41) and 0% (0/153), respectively (*p* < 0.001) (Fig. 2). Moreover, HBsAg level of > 10 IU/mL showed relatively high negative predictive value (NPV) of up to 97.9% for HBsAg loss.

### Prognostic factors for post-treatment HBsAg loss

A total of 21 patients (8.4%) achieved post-treatment HBsAg loss. We conducted Cox regression analysis to identify prognostic factors for HBsAg loss in off-treatment patients. As shown in Table 2, baseline HBsAg levels of < 25,000 IU/mL, on-treatment HBsAg decline of > 1 log10IU/mL at 24 weeks of treatment and EOT HBsAg level of ≤10 IU/mL were found significantly associated with HBsAg loss and contributive to the incidence (*p* < 0.001, *p* = 0.001 and p = 0.001, respectively).

**Table 2:** Cox regression analysis to identify prognostic factors for HBsAg loss in off-treatment patients

Variables	HR	95% CI	*p-*value
Male	1.674	0.244–11.475	0.600
Over 30 years old	0.703	0.095–5.183	0.730
Baseline ALT > 4 × ULN U/L	1.395	0.380–5.121	0.616
Baseline HBV DNA < 2 × 10^6^ IU/mL	0.097	0.007–1.379	0.085
Baseline HBsAg < 25,000 IU/mL	7.799	2.903–20.947	***< 0.001***
On-treatment HBsAg decline > 1 log_10_ IU/mL at week 24	8.329	2.470–28.087	***0.001***
On-treatment HBV DNA decline > 2 log_10_ IU/mL at week 24	12.726	0.627–258.244	0.098
Duration of IFN over 1 year	0.253	0.049–1.310	0.101
EOT HBsAg ≤10 IU/mL	7.918	2.453–25.556	***0.001***

*ALT* alanine aminotransferase*, ULN* upper limit of normal, *IFN* interferon, *EOT* end-of-treatment. *P*-values < 0.05 are shown in bold italics

### Factors affecting EOT HBsAg level

We further performed logistic regression analysis to identify patients who tend to obtain EOT HBsAg level ≤ 10 IU/mL. As shown in Table 3, high level of baseline ALT (> 4 times the upper limits of normal [ULN]), low level of baseline HBV DNA (< 2 × 106 IU/mL) and low level of baseline HBsAg (< 25,000 IU/mL) were significantly associated with relatively low EOT HBsAg of ≤10 IU/mL, moreover, HBsAg decline > 1 log10IU/mL and HBV DNA decline > 2 log10IU/mL at 24 weeks of treatment exhibited consistently statistical significance.

**Table 3:** Logistic regression analysis to identify potential factors associated with EOT HBsAg level ≤ 10 IU/mL

Variables	HR	95% CI	*p-*value
Male	0.605	0.184–1.985	0.407
Over 30 years old	0.664	0.179–2.458	0.540
Baseline ALT > 4 × ULN U/L	17.241	1.689–166.667	***0.016***
Baseline HBV DNA < 2 × 10^6^ IU/mL	7.813	1.961–31.250	***0.004***
Baseline HBsAg < 25,000 IU/mL	1.624	1.083–2.435	***0.019***
On-treatment HBsAg decline > 1 log_10_IU/mL at week 24	55.556	11.904–250	***< 0.001***
On-treatment HBV DNA decline > 2 log_10_IU/mL at week 24	14.084	3.003–66.667	***0.001***
Duration of IFN over 1 year	2.140	0.680–6.736	0.193

*ALT* alanine aminotransferase*, ULN* upper limit of normal, *IFN* interferon. *P*-values < 0.05 are shown in bold italics

### Comparison of patients with EOT HBsAg loss and in group 1

After receiving IFN-based therapy, a total of 23 patients have achieved HBsAg loss. Among them, 6 patients have achieved HBsAg loss at EOT with a average duration of 17.3 months. Each of them has received treatment of > 12 months. 17 patients in Group 1 (HBsAg ≤10 IU/mL) have achieved post-treatment HBsAg loss, 15 of them showed treatment duration of > 12 months and the average duration of the 17 patients was 14.5 months. Baseline characteristics, dynamic changes of on-treatment HBsAg and duration of IFN therapy were balanced across patients with EOT or post-treatment HBsAg loss, shown in Table 4.

**Table 4:** Comparison of patients with HBsAg loss at EOT and in Group 1

Variables	EOT HBsAg loss group	HBsAg loss in Group 1	*p-*value
Gender, male (%)	5 (83.3%)	12 (70.6%)	0.541
Age (years)	24.8 ± 2.7	26.5 ± 5.7	0.496
Baseline ALT (U/L)	249.8 ± 214.0	133.6 ± 130.1	0.127
Baseline HBV DNA (log_10_IU/mL)	4.3 ± 1.5	5.8 ± 2.1	0.133
Baseline HBsAg (log_10_IU/mL)	2.6 ± 0.9	2.9 ± 0.6	0.399
On-treatment HBsAg decline at week 24 (log_10_IU/mL)	1.6 ± 1.4	1.2 ± 0.8	0.37
On-treatment HBV DNA decline at week 24 (log_10_IU/mL)	1.9 ± 1.6	3.7 ± 1.6	***0.041***
Duration of IFN (months)	17.3 ± 4.9	14.5 ± 5.2	0.254

*P*-value < 0.05 is shown in bold italics

## Discussion

In this retrospective observational study, we demonstrated that in HBeAg positive patients treated with IFN, patients with EOT HBsAg ≤200 IU/mL, especially ≤10 IU/mL, might achieve better post-treatment response after cessation of therapy. Among all patients, HBsAg loss at 5 years post-treatment was achieved by 21 (8.4%) individuals, and 17 belonged to group 1 (30.4%), 4 belonged to group 2 (9.7%). HBsAg reversion was not observed during the whole follow-up. The post-treatment response to IFN observed in this study is consistent with Chuang et al. and Marcellin et al. reports [11, 16].

At present, the response rate of IFN therapy is not satisfactory. Pretreatment HBsAg, viral load and ALT levels were highlighted to predict post-treatment response of IFN therapy. For CHB patients with pretreatment high ALT level, low viral load and low HBsAg level, treatment with IFN leads to better clinical outcomes [24–27]. Consistent with previous studies, in our study, we found that baseline ALT > 4 × ULN, HBV DNA < 2 × 106 IU/mL and HBsAg < 25,000 IU/mL were significantly associated with EOT HBsAg ≤10 IU/mL. Likewise, patients with HBsAg decline > 1 log10IU/mL and HBV DNA decline > 2 log10IU/mL at 24 weeks of treatment were likely to achieve HBsAg ≤10 IU/mL at EOT, consistent with previous reports that response-guided treatment adjustment (the RGT rule) might optimize the on-treatment and off-treatment management [16–21]. Furthermore, we demonstrated that, pretreatment low level of HBsAg, HBsAg decline > 1 log10IU/mL at 24 weeks of treatment and EOT HBsAg ≤10 IU/mL were associated with post-treatment HBsAg loss, indicating that on-treatment response can also predict serological response of long-term follow-up after treatment cessation. We also demonstrated that EOT HBsAg level can serve as prognostic factor for post-treatment response to IFN. EOT HBsAg ≤10 IU/mL was found to be a protective factor, and a HBsAg level > 10 IU/mL can serve as a satisfactory negative predictor for post-treatment HBsAg loss (NPV of 97.9%). Thus, for patients with EOT HBsAg level > 10 IU/mL, cessation of treatment may not be recommended. Furthermore, it has been reported that the extended IFN treatment improves the outcome of HBeAg-negative patients [28]. In a clinical trial with relatively small sample size, extending the duration of IFN to 60 weeks has been found to result in a higher rate of sustained virological response, and 5 of the total 13 patients showed a > 90% decrease in HBsAg concentration after 60-week IFN treatment [29]. The consensus on pegylated interferon (Peg-IFN) reported that for the patients with undetectable HBV DNA and low HBsAg level (< 10 IU/mL) at 48 weeks of Peg-IFN treatment, extended treatment to 72 weeks or even longer should be considered to achieve ideal treatment endpoint, HBsAg clearance [14]. However, the treatment gap between low HBsAg level (≤10 IU/mL) and HBsAg loss, also known as functional cure, is volatile and rather long for some of the patients. Extending the Peg-IFN treatment to 72 or 96 weeks, can not guarantee HBsAg loss for the majority of CHB patients. In this study, there were 30.4% patients who have achieved HBsAg loss in Group 1, and none of them exhibited HBsAg reversion during the whole follow-up. In addition, we demonstrated that the duration of IFN therapy was not associated with low EOT HBsAg level (≤10 IU/mL) and post-treatment HBsAg loss. Hence, off-treatment follow-up could be considered for patients achieving HBsAg level ≤ 10 IU/mL with inadequate economic conditions and unwillingness to extend treatment. While in Group 2, only 9.7% patients achieved HBsAg loss. Thus, for patients with HBsAg level of > 10 and ≤ 200 IU/mL at EOT of IFN, extended treatment to achieve HBsAg decline to ≤10 IU/mL is necessary before off-treatment follow-up.

We further analyzed clinical characteristics of the 17 patients who obtained EOT HBsAg ≤10 IU/mL and achieved post-treatment HBsAg loss. And we found that the distribution of baseline characteristics, dynamic changes of on-treatment HBsAg and duration of IFN therapy were balanced across patients with EOT or post-treatment HBsAg loss, indicating that EOT HBsAg ≤10 IU/mL can serve as satisfactory end-point of treatment. Recent researches concerning off-treatment HBsAg loss to NUCs therapy, have reported that both EOT HBsAg level < 200 IU/mL [30, 31] and < 10 IU/mL [32] are important contributing factors in achieving off-treatment HBsAg loss. However, these studies mainly refer to virological and clinical relapse, whether HBsAg reversion occurred during follow-up is not mentioned. In this study, patients in Group 1 who achieved post-treatment HBsAg loss did not exhibit HBsAg reversion. The possible explanation is that IFN can suppress covalently closed circular DNA (cccDNA) in hepatocytes and modulate host immune response, the indirect antiviral effects of IFN lead to sustained immune control after treatment discontinuation [9, 10].

There were notable limitations to this study. First, since the retrospective design nature of our study, some indicators, including HBV genotype, HBeAg titer, liver fibrosis stage, treatment experience with prior IFN or NUCs, will inevitably be missing, and this study is biased to a certain extent. However, we enrolled a large sample of patients to minimize this limitation and should be valuable to other investigators and clinicians. Second, our study included HBeAg-positive patients only and the results were not applied for HBeAg-negtive patitents. As we know, immune status in HBeAg-positvie is entirely different to HBeAg-negative patients which need further research. Third, HBsAg levels of treatment week 12 was not available in our study due to the incomplete data.

## Conclusions

In conclusion, besides baseline HBsAg level and on-treatment dynamic changes of HBsAg level, we identified EOT HBsAg level as a monitoring indicator for IFN therapy. EOT HBsAg level of ≤10 IU/mL was found to lead to high rate of off-treatment HBsAg loss. For patients without willingness to extend IFN treatment, off-treatment follow-up could be considered when HBsAg level decreased to ≤10 IU/mL.

## Data Availability

The datasets used and/or analysed during the current study are available from the corresponding author upon reasonable request.

## Abbreviations

CHB: Chronic hepatitis B
IFN: Interferon
HBsAg: Hepatitis B surface antigen
EOT: End-of-treatment
NPV: Negative predictive value
HBV: Hepatitis B virus
HCC: Hepatocellular carcinoma
NUCs: Nucleos(t) ide analogues
HBeAg: Hepatitis B e antigen
ALT: Alanine aminotransferase
SD: Standard deviation
ULN: Upper limits of normal
cccDNA: Covalently closed circular DNA
